# A hybrid implementation-effectiveness study of a school-based intervention for promoting health and well-being in low-resource settings: the ISOBAR study protocol

**DOI:** 10.3389/fpsyt.2026.1823889

**Published:** 2026-07-14

**Authors:** Swaran P. Singh, Catherine Winsper, Nadia Binte Alam, Tolulope Bella-Awusah, Graeme Currie, Oluwabunmi (Tokun) Fola-Bolumole, Domenico Giacco, Paramjit Gill, Sanjana Goutham, Srividya N. Iyer, Jasmine Kalha, Isha Lohumi, Jason Madan, Dafne Moroni, Olayinka Omigbodun, Soumitra Pathare, Shiva Prakash Srinivasan, Simon Smith, J.S. Thakur, Thara Rangaswamy, Helena Tuomainen, Samuel Watson, Sagar Jilka

**Affiliations:** 1 Health Sciences, Warwick Medical School, Warwick University, Coventry, United Kingdom; 2 Coventry and Warwickshire Partnership National Health Service (NHS) Trust, Coventry, United Kingdom; 3 Department of Psychiatry and Centre for Child and Adolescent Mental Health, College of Medicine, University of Ibadan, Ibadan, Nigeria; 4 Warwick Business School, Warwick University, Coventry, United Kingdom; 5 Department of Psychiatry, McGill University, Montreal, QC, Canada; 6 Centre for Mental Health Law and Policy, Indian Law Society Pune, Pune, India; 7 Schizophrenia Research Foundation, Chennai, India; 8 Centre of Excellence for Evidence Based Research on Non-Communicable Disease (NCDs) in Low- and Middle-Income Country (LMICs), World Non-Communicable Disease (NCD) Federation, Chandigarh, India; 9 Child and Adolescent Mental Health School of Nursing and Midwifery University of Worcester, Worcester, United Kingdom; 10 Department of Applied Health Sciences, University of Birmingham, Birmingham, United Kingdom; 11 Warwick Centre for Global Health, University of Warwick, Coventry, United Kingdom

**Keywords:** adolescents, health literacy, implementation science, mental health, schools

## Abstract

**Introduction:**

School-based interventions can improve adolescent health outcomes and tackle the growing burden of non-communicable diseases (NCDs) in low-and-middle-income countries (LMICs). Results to date are variable, partly due to lack of cultural adaptation of Westernised models and limited focus on implementation processes. The aim of the ISOBAR project is to develop, implement, and test a school-based intervention to address the emergence of mental and physical (i.e., nutritional, physical inactivity) health problems in LMICs. The three-stage intervention comprises (1) assessment for mental and physical health problems, (2) universal health literacy, and (3) indicated counselling.

**Methods and analysis:**

The ISOBAR multi-site project comprises three phases: (1) pre-intervention (co-development and cultural adaptation of the intervention); (2) implementation of the intervention; and (3) post-intervention evaluation. The intervention will be delivered using a staggered roll-out design to introduce the intervention sequentially to three schools per site (nine in total) in Chennai, Gujarat (India), and Ibadan (Nigeria). Each school will receive the intervention by the end of the study period. Local teams will recruit 200 adolescents per school (Total n=1, 800). All adolescents (in intervention and control conditions) will be assessed for mental health/behavioural problems and nutritional/weight problems at baseline. Adolescents in the intervention school will receive the universal health literacy intervention (main cohort), and those adolescents reaching pre-determined thresholds on mental health and/or nutritional indices (sub-cohort) will be referred to school-based counselling support. A suite of assessments will be conducted throughout the study period including: (1) intervention effectiveness (e.g., impact on help-seeking, weight, and mental health and behavioural outcomes); (2) implementation processes (e.g., facilitators and barriers) and outcomes (e.g., acceptability, appropriateness, sustainability); and (3) cost-effectiveness.

**Ethics and dissemination:**

The study was approved by the University of Warwick’s Biomedical and Scientific Research Ethics Committee (BSREC 36/23-24) and the institutional ethics committees of all participating sites. Research findings will be disseminated through peer reviewed scientific publications, public announcements in local communities, policy briefings, print and online media, and institutional and professional social media accounts and websites.

## Highlights

- The ISOBAR study will develop a bespoke three-stage composite school-based physical and mental health intervention, which is culturally adapted to each study region.
- Intervention development and delivery is grounded in co-production to ensure suitability to setting and recipients.
- ISOBAR will build research capacity for future studies in LMICs including in implementation science and health economic evaluation.
- Due to logistical and ethical considerations, we are unable to conduct a randomised controlled trial meaning causal inference will be limited.
- For ethical reasons, students in the control condition will be provided guidance and signposting to local services, which might ‘dilute’ the effect of the intervention.

## Introduction

Adolescence (12–18 years) is a critical transition period of significant psychological, physical, emotional, social, and cognitive growth (1), with heightened risk of internalising (e.g., depression, anxiety) and externalising (e.g., conduct problems, substance misuse) problems (2). Most non-communicable diseases (NCDs), such as diabetes and depression, are associated with lifestyle habits and behaviours established during childhood and adolescence (3). NCDs are a growing burden in low-and-middle income countries (LMICs), affecting individuals at a younger age and leading to worse outcomes than in high income countries (HICs) (4). The scope of prevention programmes is incommensurate with the health burden of NCDs in LMICs (5) highlighting the need for early intervention to address physical and mental health problems (e.g., physical inactivity, substance misuse, diet) concomitantly.

Adolescents in LMICs face limited access to healthcare due to multiple barriers across dimensions including geographical accessibility, availability, affordability, and acceptability (6). School programmes can play a key role in tackling NCDs by promoting healthy behaviour (e.g., improved diet and activity) and increasing knowledge about maintaining psychological well-being and help-seeking. Schools provide an ideal setting for reaching a large number of adolescents (7) through the delivery of multi-component interventions to address physical and mental health outcomes simultaneously. Multi-component interventions combine two or more distinct, evidence-based therapeutic or behavioural components such as exercise, education and medication to address complex health issues (8). Such multi-component interventions are especially attractive in LMICs as they can have broad effects for the cost of a single programme, and are less likely to be “pushed out” if policy priorities change (9).

Reviews on school-based interventions in LMICs cover a range of continents and sub-continents including Asia, Africa, and Latin America (10–13). Interventions tend to focus on either mental (10, 11) or physical (14, 15) health outcomes and are typically delivered at one of three levels: (1) universal (to all students); (2) selective (to at-risk students); or (3) indicated (to students with elevated symptoms) (16). Most universal interventions aim to enhance knowledge about nutrition, physical health and activity, mental health and illness, hygiene, and reproductive and sexual health (10, 17). Some mental health interventions address life skills including resilience, self-efficacy, emotional well-being, and interpersonal communication (13). Others include targeted approaches for at-risk or sub-clinical youth, such as additional activity sessions for obese students or narrative therapy for traumatised children (16). Missing from the literature are studies of composite school-based programmes integrating physical and mental health components, and universal (e.g., health promotion) with indicated (e.g., counselling or therapy) approaches to ensure that adolescents in need are encouraged to seek help.

School-based interventions developed in HICs cannot be simply transported to LMIC contexts due to profound differences in infrastructure, resources, cultural beliefs, and traditions. Although RCTs have been conducted successfully in LMICs, explanatory trial conditions may not capture key implementation challenges such as resource constraints, workforce capacity, school timetables, governance arrangements, and local referral pathways.

Schools are multi-level implementation contexts with many priorities, decision-makers (e.g., governing bodies, parent organisations), and recipients (e.g., students, parents, teachers) (18). Thus, implementation barriers can exist at individual (19), organisational (20), and community (10) levels. Although there is evidence of adapted and implemented single-component school-based interventions in LMICs, we lack detailed information on how to do this for multi-component interventions integrating both mental health support and nutritional health literacy. This signals the need for pragmatic implementation studies to ensure that interventions can be sustained outside of research settings and scaled up at country level (20).

### Objectives, aims, and research questions

The main aim of ISOBAR is to develop and test the effectiveness and implementation of a school-based three-stage (assessment and referral, universal health literacy, and indicated counselling) composite physical and mental health intervention in India (Chennai and Gujarat) and Nigeria (Ibadan).

Key objectives include:

1. To co-develop and culturally adapt a universal health literacy intervention combining mental and physical health modules.
2. To assess all adolescents for emotional, behavioural, and nutritional/weight problems using validated assessment tools.
3. To develop, and culturally adapt, an indicated counselling intervention for adolescents reaching pre-determined thresholds on the above universal assessment indices.
4. To implement and evaluate the school-based intervention at each site using quantitative and qualitative implementation assessment tools.
5. To assess the costs of the intervention and explore how these relate to consequences and affordability.
6. To estimate the effectiveness of the school-based intervention (health literacy and counselling components) with a suite of outcome measures, including help-seeking, mental health symptoms, diet and activity, and anthropometric measures.

We have two primary research questions:

1. Does a health literacy intervention (delivered to all adolescents at classroom level) increase help-seeking behaviours?
2. Do adolescents who receive counselling for physical and/or mental health problems experience improved outcomes?

## Methods and analysis

ISOBAR is an implementation-effectiveness study (21) designed to investigate the cultural adaptation, implementation, and outcomes of a school-based composite intervention at three different sites in India and Nigeria. The study commenced in October 2023 and will conclude in October 2027.

### ISOBAR programme

The ISOBAR programme is a school-based intervention which comprises three components: (1) universal assessment and referral; (2) universal health literacy; and (3) school-based counselling for adolescents exceeding pre-defined thresholds. It will be delivered to adolescents aged 13–18 years old, by local counsellors paid and employed to exclusively work on the study.

### ISOBAR programme management

The ISOBAR research programme brings together seven institutions in the UK, India, Nigeria and Canada; institutional roles are described in the **Supplementary Material**. The University of Warwick was the sponsor organisation.

We will use Open Data Kit (ODK) for the centralised management of all study data (22). ODK is a system for facilitating easier collection of data using mobile devices such as tablets and smartphones. It is open source and therefore freely distributed and makes the transition from paper to digital surveys quick, allowing large amounts of data collection with a mobile device. This system allows for built-in checks to facilitate better quality data, which can be collected offline and encrypted at source. It also allows for the monitoring of data collection remotely including the ability to make changes once data collection has started. Each study site will upload data onto the ODK system weekly, which will be quality checked during regular cross-site data management meetings.

### Study sites

Our three study sites are situated within India (Chennai and Gujarat) and Nigeria (Ibadan), which both have a large adolescent population and a severe shortage of mental healthcare provision. Chennai is the capital of the state Tamil Nadu and is one of the major metropolitan cities in South India with a fast growing, cosmopolitan population. Tamil Nadu is the 7^th^ most populous state in India (population: 81 million) and has approximately 0.3 psychiatrists and 0.04 clinical psychologists per 100,000 people (23). Gujarat is India’s 9^th^ most populous state with a population of approximately 69 million. Gujarat has approximately 0.31 psychiatrists, 0.03 clinical psychologists and 0.02 psychiatric social workers per 100,000 people (24). Ibadan is the capital city of Oyo State, which is the 6^th^ most populous state in Nigeria (population: 8 million). We do not have exact figures on the number of mental health professionals per 100,000 people in Oyo State; however, figures for Nigeria indicate a very low ratio with approximately 0.09 psychiatrists and 0.02 clinical psychologists and social workers per 100, 000 people (25).

### ISOBAR programme design

ISOBAR is a type 2 hybrid effectiveness-implementation study, with a dual focus on effectiveness and implementation outcomes (26). It comprises three main phases: (1) pre-intervention; (2) implementation of intervention; and (3) post-implementation evaluation.

### Phase 1: pre-intervention stage

The key aims of the pre-intervention stage were to: (a) develop and pilot a school-based intervention comprising a universal health literacy component and an indicated counselling component for adolescents who exceed threshold symptoms; (b) ensure that the intervention is culturally adapted to each study site; (c) develop relationships with local schools, parents, and advisory boards to facilitate implementation; (d) recruit and train counsellors to deliver the intervention; and (e) pilot universal assessment tools.

### Intervention development and adaptation

**Figure 1:**
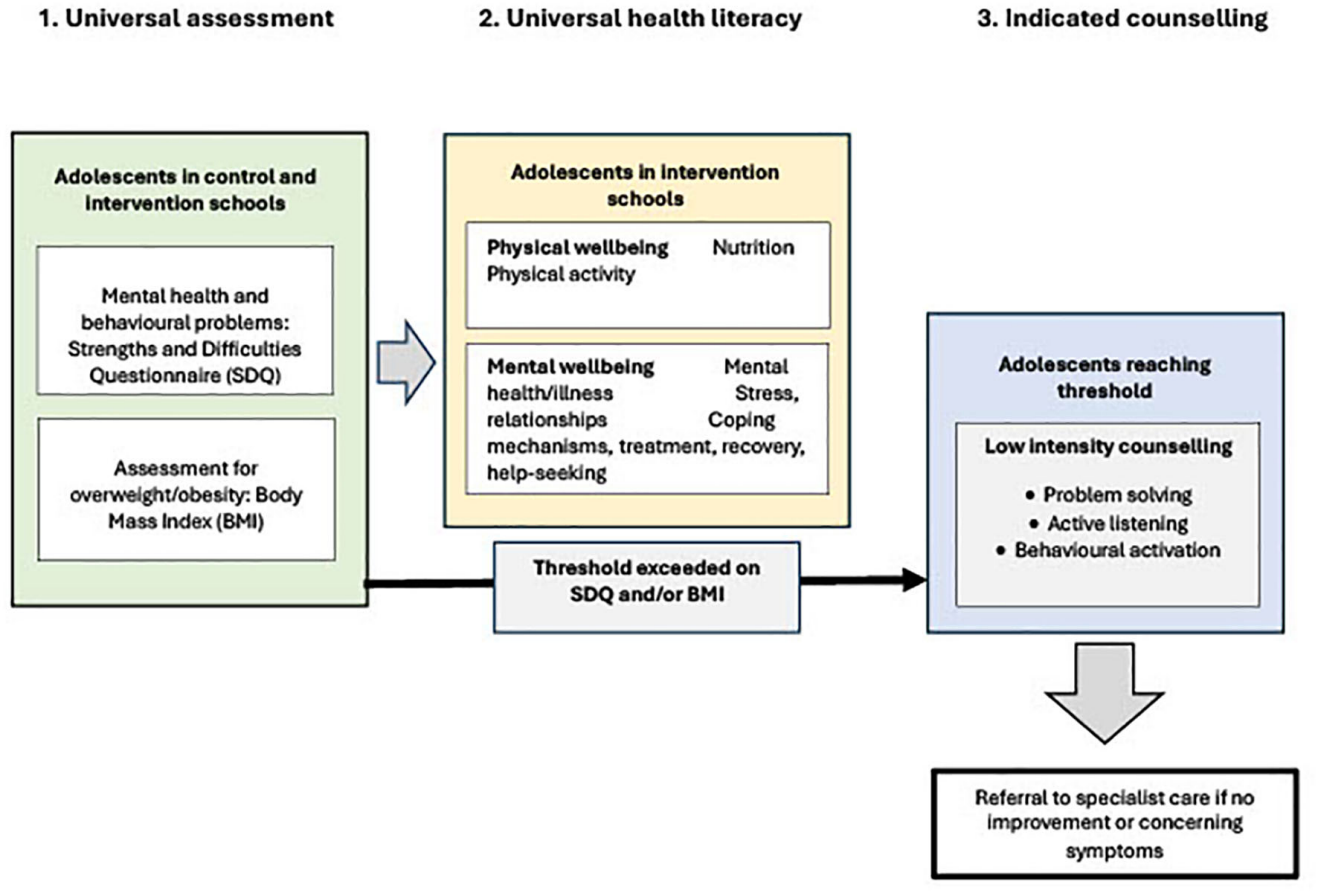
The three components of the school-based intervention.

The school-based intervention comprises three components: (1) universal assessment and referral; (2) universal health literacy; and (3) school-based counselling for adolescents exceeding pre-defined thresholds (**Figure 1**).

The intervention will be developed using an evidence-informed and iterative approach, drawing on existing frameworks and relevant literature on school-based adolescent health interventions. Local teams conducted country-specific rapid literature reviews to identify locally developed and tested interventions that address components of mental, physical, or nutritional health literacy in their respective countries. The focus was on identifying a selection of key evidence-based components relevant to adolescent mental health and health promotion in school settings, particularly in low- and middle-income contexts.

Specifically, the reviews highlighted the effectiveness of:

- Mental health literacy interventions, including recognition of common mental disorders, stigma reduction, and promotion of help-seeking
- School-based psychosocial interventions, particularly those incorporating coping skills and problem-solving approaches
- Nutrition and physical activity interventions, including behavioural change strategies and school-based health promotion
- Participatory and skills-based approaches, such as interactive learning and practical components

These findings guided the structure and content of the intervention, as well as implementation determinants (e.g., delivery methods, cultural adaptations, facilitators and barriers).

Patient and public involvement (PPI) will be embedded across multiple phases of the study. Relevant stakeholders will be engaged throughout intervention development, refinement, and implementation to ensure that the intervention is contextually appropriate, acceptable, and feasible. Their role will include contributing to the co-production of the intervention, providing feedback during iterative refinement, and supporting implementation through ongoing engagement and advisory input. This approach is intended to enhance relevance, feasibility, and potential for sustainability. Intervention development workshops will be conducted at each site using co-design principles and methodology to gather stakeholder (adolescents, parents, teachers) input on intervention content and structure. Iterative review will be used to ensure contextual relevance, acceptability, and feasibility within the study setting.

Local teams conducted country-specific rapid reviews to identify effective elements (e.g., content, delivery methods) and implementation determinants (e.g., cultural adaptations, facilitators and barriers) of school-based physical and mental health interventions. These reviews were supplemented with a global systematic umbrella review to collate evidence on common components, mechanisms, moderators, and outcomes across interventions in LMICs (currently under review). The literature reviews underpinned the intervention development workshops conducted at each site. These workshops were recorded and transcribed to ensure accurate capture of the co-production process. Local teams will continue to monitor and document adaptation and implementation using field logs.

#### (1) Universal assessment and referral

We selected assessment tools to measure mental health and behavioural problems (Strengths and Difficulties Questionnaire: SDQ; Global School-Based Student Health Survey: GSHS), well-being (WHO-5 well-being index), nutritional problems, and at-risk weight status (Body Mass Index: BMI) Tools selected were: (1) relatively quick to administer; (2) well-established; (3) previously used and validated in our study countries; and (4) available in the local language. We will also assess help-seeking behaviour and health literacy with bespoke scales. The health literacy scale will draw on the conceptual framework developed by Jorm (27) and capture key components of the intervention (e.g., beliefs about the utility of counselling, stigma surrounding mental illness). Assessment scales are self-report and will be completed by adolescents at baseline, 8, 15 and 22 months with support from trained research assistants. Weight, height and arm circumference measurements will be recorded by researchers. All adolescents (in control and intervention schools) will be assessed. Adolescents in the intervention school reaching the pre-determined threshold on either the SDQ and/or the BMI (**Table 1**) will be referred to the inhouse counselling intervention. Adolescents in the control schools who are identified through the universal assessments as having potential mental health or nutritional concerns will not be left without support. All participants with such concerns will be informed of their screening results in an age-appropriate manner. Where necessary, efforts will be made to inform parents or guardians through the school system, in line with ethical and safeguarding procedures. Participants and their families will be advised to seek appropriate care, and the research team will facilitate this by providing information on available services, including referral pathways to specialist mental health care.

**Table 1:** Universal assessments administered to all adolescents in intervention and control schools.

Name of scale/assessment	Description	Items in scale	Threshold for referral to counsellor	Time-points
Socio-demographic questionnaire	Bespoke scale assessing key demographic factors (culturally adapted for each site)	Age, gender, religion, parents’ education level, family size and composition, geographical setting	N/A	Baseline
Help-seeking behaviour questionnaire	Bespoke scale assessing help- seeking	Sought help in the past six months, frequency of help- seeking, and who from/what for	N/A	Baseline, 8, 15, 22 months
Health literacy	Bespoke scale based on Jorm (27) conceptual framework	Example items: Ability to give advice, how to improve well-being, thoughts on counselling, stigma, knowledge of mental illness	N/A	Baseline, 8, 15, 22 months
Strengths and Difficulties Questionnaire (SDQ)	Validated scale to assess emotional and behavioural issues	• Emotional symptoms • Conduct problems • Hyperactivity/inattention • Peer problems • Prosocial behaviour	• Based on algorithm (hierarchical): -Very high score/very high impact - High score/very high impact -Very high score/high impact -High score/high impact (Impact score is given more weight)	Baseline, 8, 15, 22 months
WHO-5 well-being index	Validated measure of well-being	• Cheerful/good spirits • Calm and relaxed • Active and vigorous • Fresh and rested • Things that interest me	N/A	Baseline, 8, 15, 22 months
Global School-Based Student Health Survey (GSHS)	Validated scale for adolescents to assess risks for morbidity and mortality	• Dietary behaviours • Drug use • Hygiene • Mental health • Physical activity • Sexual behaviours • Drug use • Tobacco use	N/A	Baseline, 8, 15, 22 months
Body Mass Index (BMI)	Screening tool for weight-related health risks	• Weight in kg • Height in metres^2^	Sites will follow country specific thresholds for overweight and obesity	Baseline, 8, 15, 22 months

#### (2) Universal health literacy

The health literacy component is designed to improve knowledge, attitudes, and behaviours relating to mental well-being, nutrition, and physical health. It will be targeted at adolescents (e.g., classroom didactic and activity sessions, role playing, homework), parents (e.g., meetings) and school environments (e.g., improvements to school aesthetics, topics presented in assemblies). Each site has produced a bespoke health literacy training manual to guide counsellors in delivery. Manuals were developed in consultation with professionals in mental and nutritional health and draw on existing programmes from respective countries, e.g., the School Health and Wellness Programme in India (28), UNFPA’s Action for Adolescent Girls in Nigeria (29).

Manuals across sites share common content but also have variations (e.g., topics covered such as gender identity in India, and provision of breakfast and vegetable gardens in Nigeria, length and frequency of sessions) based on cultural adaptations and stakeholder engagement work. Common content across health literacy manuals includes: (1) understanding physical health (e.g., importance of physical activity, lifestyle diseases); (2) understanding the relationship between mental health and mental illness, and mental health and physical health; (3) nutrition for adolescents; (4) understanding stress, relationships, and common changes during growth; (5) common mental disorders in young people; and (6) coping mechanisms, treatment, recovery, and help-seeking.

#### (3) Indicated counselling

All students identified as having serious or very high emotional or behavioural problems, with functional limitations as measured by the SDQ, and those children who did not screen positive on the SDQ but sought help, will be provided with in-school counselling within a month of the initial assessment. Sites are currently developing and piloting site-specific manuals to underpin the counselling component of the intervention. The manual will assist with the training of the counsellors and help ensure fidelity. Counsellors will be encouraged to develop a system of peer support across sites to facilitate mutual learning and enhance problem-solving skills. Counsellors will be trained to: (1) support young people with nutritional and/or mental health problems; (2) identify serious problems requiring specialist care and help young people to access that care; and (3) support teachers in schools to refer adolescents in need. Counsellors will use various techniques including problem-solving, active listening, and behaviour activation. Format and duration of counselling sessions will be tailored to adolescent need and severity of symptoms, with (one-to-one or group) sessions available for the duration of the project (or as needed) once the counselling is introduced into the intervention school). Adolescents will be referred for specialist care (e.g., psychiatric care) if there is no improvement during the counselling intervention period, or if the adolescent is demonstrating psychotic symptoms or suicidal ideation. Three counsellors per site will be recruited to deliver the intervention. Chennai and Gujarat will recruit graduates from mental health and social work fields as counsellors. Ibadan will recruit lay health workers due to a shortage of mental health professionals in the area.

### Phase 2: implementation of the intervention

**Figure 2:**
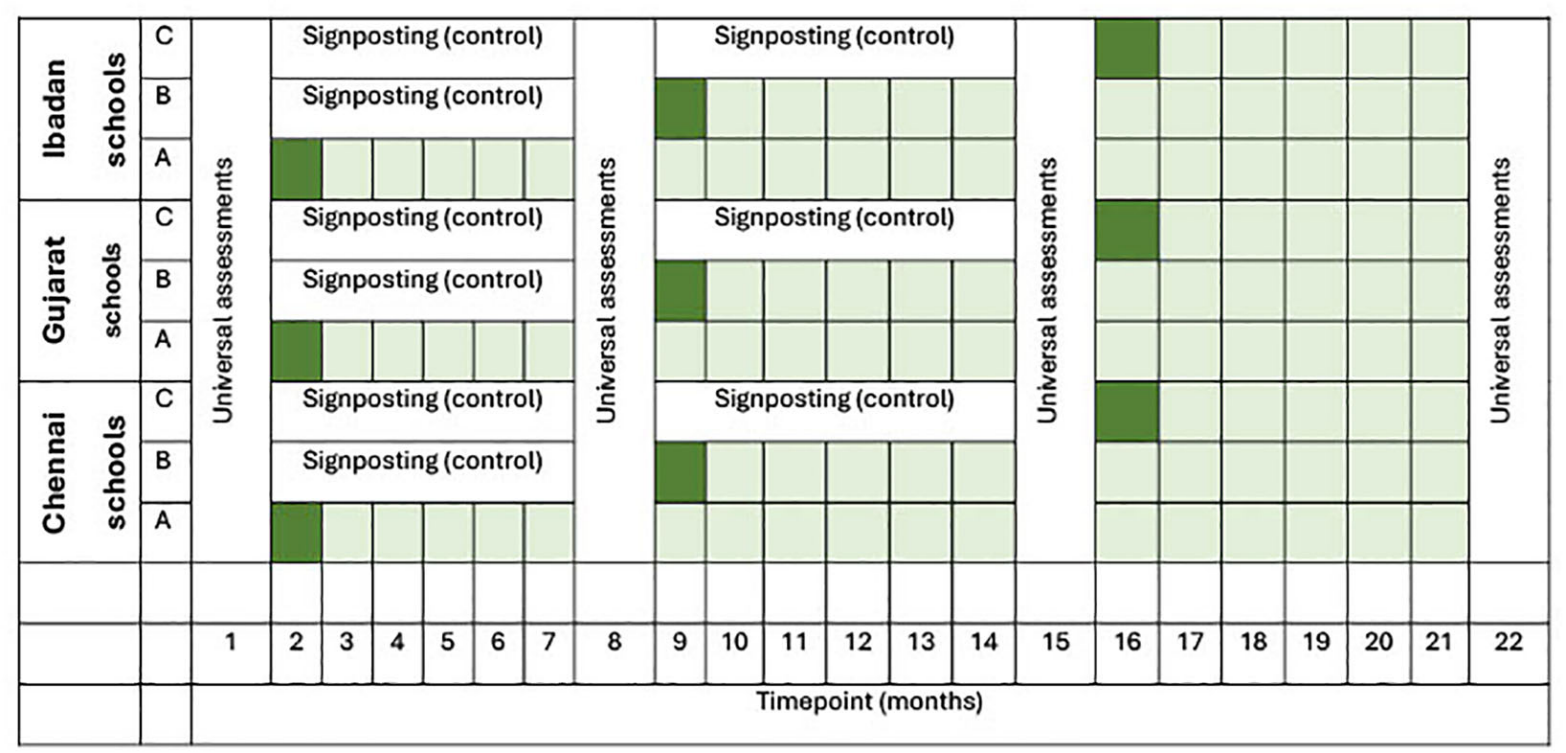
A schematic view of intervention delivery at Chennai, Gujarat, and Ibadan. There are three schools (A, B, C) per site; universal assessments are conducted at four time-points (baseline, 8 months, 15 months, 22 months); dark green box represents delivery of universal health literacy; light green box represents delivery of counselling intervention.

This is an implementation study, not a trial. It is funded to study the implementation of an intervention with proven effectiveness in another setting, distinct from where the effectiveness trial was conducted. We will adopt a non-randomised staggered roll-out design, informed by stepped wedge principles (30), as outlined in **Figure 2**. Outcomes will be collected in all schools at baseline and at each subsequent assessment point, while schools transition sequentially from control to intervention conditions. Because there are only nine schools across three sites, randomising the order of roll-out would add little to statistical inference; instead, the order will be determined pragmatically according to school readiness, logistical feasibility, and local implementation requirements. The main implication is that causal inference will be more limited than in a randomised stepped wedge trial, and findings will therefore be interpreted alongside school and time fixed effects and the accompanying implementation evaluation. The staggered roll-out design ensures that each school will receive the intervention at a different time point and that all schools will receive the intervention by the end of the programme. We have opted for this approach for the following reasons. First, It is an implementation study. Second, since the intervention is likely to have beneficial effects, it is unethical to withhold it from schools that would form the control groups. Finally, logistically, we cannot implement the intervention in all participating schools at the same time. Staggering the intervention over time allows for our hub-and-spoke model of counsellors to offer the intervention to all students in need at each school.

The intervention will be delivered in two stages: (1) all adolescents in the intervention school will receive the health literacy component (dark green box); (2) a subset of adolescents in the intervention school will receive the counselling component if they meet the pre-defined threshold indicating nutritional/weight and/or mental health problems (light green box). These adolescents will comprise the sub-cohort who will be followed up at two/three and 12 months. Once the counsellors are introduced into each school, they will remain for the duration of the study.

Each study site will have three schools (school A, B and C) who will receive the intervention in a staggered manner so that each of the three schools will eventually receive the intervention. Schools will initially serve as the control condition and then incrementally transition to the intervention phase. Therefore, while schools function as the control condition at specific time points, there are no fixed “control schools.” We do not consider contamination to be an issue in this design because students will not switch schools, and counsellors are assigned to a school each.

In intervention schools, children identified with mental or physical health challenges will be referred for further evaluation and care by the school counsellor, with support from the school point of contact. In schools that are still in the control condition, information about children with identified mental or physical health challenges will be shared with the school point of contact, who will inform family members as needed and provide signposting advice (e.g., posters and announcements about local services and helplines). All adolescents in the intervention and control conditions will complete the baseline universal assessments prior to roll-out of the intervention. Adolescents in the sub-cohort will complete targeted assessments (**Table 2**) at baseline, two/three and 12-month follow-up. All selected schools are publicly funded and run by the Government. We will provide training to teachers within the selected schools (e.g., identification of symptoms and referral pathways).

**Table 2:** Targeted assessments administered to cohort of adolescents receiving school counselling support.

Name	Description	Items in scale	Time-points
Patient Health Questionnaire (PHQ-9)	Validated depression scale	• Little interest or pleasure • Feeling down/hopeless • Trouble sleeping • Feeling tired • Poor appetite/overeating • Feeling bad about self • Trouble concentrating • Moving/speaking slowly • Thoughts be better off dead	Baseline, 2 and 12 months
Generalised Anxiety Disorder scale-7 (GAD-7)	Validated scale for anxiety symptoms	• Feeling afraid • Becoming annoyed/irritable • Being restless • Trouble relaxing • Worrying too much • Not able to stop/control worry • Feeling nervous/anxious	Baseline, 2 and 12 months
Childrens Global Assessment Scale (C-GAS)	Validated scale for adolescent functioning	General functioning is assessed based on information from previous assessments and interviews with the child, their parents, carers, and school.	Baseline, 2 and 12 months
The SDQ follow-up questions	Questions to address whether intervention has helped mental and behavioural health	• Has the intervention reduced problems? • Has the intervention helped in other ways, e.g. making the problems more bearable?	2 and 12 months
Physical health and diet questions (selected from existing scales)	Bespoke questions to address whether intervention has helped physical health and diet (items taken from validated scales)	Example items: • Exercise can help me reduce my stress level • I know what healthy food products are • If I eat five or more fruit/veg a day, I will feel good about myself	2 and 12 months

School counsellors will be responsible for delivering all stages of the intervention, with some assistance from project experts in Ibadan. We will ensure that elements of the study (including assessments and intervention modules) are tailored to routine school activities where possible to minimise burden on schools and maximise implementation success (e.g., some health literacy modules will be presented during assembly, weight measurements will be taken as part of school’s regular health monitoring schedule). There will be some flexibility incorporated into individual site timelines to account for variations in holidays and exam periods (as discussed with stakeholders during the development work).

### Participants and recruitment

We will recruit adolescents from three schools per site (total = nine schools). The inclusion criteria are: (1) being a school going adolescent aged 12–18 years; and (2) willing to take part in the mental and physical health assessments. We will recruit approximately 200 adolescents per school, equalling 600 adolescents per site, and 1, 800 adolescents in total. In India, adolescents will be recruited from grades 6 to 8. Pilot work in Chennai and Gujarat indicates that this will equal approximately 200 adolescents per school. In Ibadan, adolescents will be recruited using stratified random sampling to reach the sample target number. Sites will continually monitor intervention roll out to allow improvements to be incorporated in real-time. Implementation strategies will include providing ongoing training and supervision for counsellors, local consensus discussions and educational meetings, creating a learning collaborative approach to capture and share local knowledge, and auditing the implementation processes to enhance participation and maintenance.

### Phase 3: post-intervention phase

Assessments conducted at the post intervention phase include: (a) evaluation of the implementation process; (b) impacts of the health literacy and counselling components of the intervention; and (c) health economic evaluation.

### Evaluation of implementation process

Guided by the Consolidated Framework of Implementation Research (CFIR) (31), we will assess a comprehensive range of implementation outcomes using both quantitative and qualitative tools (32). Our primary quantitative outcomes (**Table 3**) are acceptability and appropriateness, which we will assess using published scales (e.g., Acceptability of Intervention Measure) and screening forms (e.g., percentage of teachers trained, percentage of adolescents engaging with the intervention). Secondary quantitative outcomes include fidelity, adoption and sustainability which will be assessed with published scales (e.g., NoMAD instrument) and screening forms (e.g., percentage delivery and adherence, number of sites continuing the intervention). We will also assess implementation outcomes at the school (e.g., reach and equity) and individual (e.g., adolescent satisfaction) level including whether there are differences in implementation outcomes based on gender and other social determinants.

**Table 3:** Main quantitative implementation outcomes.

Implementation outcome	Description	Published scales	Screening forms
Acceptability	Is the intervention acceptable in terms of content, delivery, complexity and credibility	Acceptability of Intervention Measure (AIM) • Meets approval • Appealing to me • Likes • Welcome	• % Teachers trained in care pathway guidance • % Adolescent participation and engagement
Appropriateness	Is the intervention compatible with the setting and suitable for adolescents	Intervention Appropriateness Measure (IAM) • Seems fitting • Seems suitable • Seems applicable • Seems a good match	N/A
Fidelity	Was the intervention implemented as intended	N/A	• % Delivery and adherence to each component • Number of dropouts
Adoption	Extent of uptake of the intervention	Normalisation Measure Development Questionnaire (short NoMAD) Example items: • I can see the potential value of [the intervention] for my work • There are key people who drive [the intervention] delivery forward and get others involved	N/A
Sustainability	Intervention and the benefits it will generate are sustained overtime Extent to which adolescents who required specialist care continued treatment over time	Normalisation Measure Development Questionnaire (short NoMAD) Example items: • I will continue to support [the intervention]	• Number of sites that continue the intervention past the study • % sustained personalised treatments

Our quantitative assessments will be supplemented with in-depth qualitative interviews and focus groups with teachers, families, adolescents, counsellors, and policy makers. We will consider the impact of internal and external context on intervention development, delivery, and reception. External context might include factors such as government policy (or lack of), financial resource, workforce capability, and cultural assumptions (such as stigma and extended family ethos and support). Internal context could include factors such as leadership (e.g., accountability, influence), background of counsellors, knowledge exchange and capacity building, co-production and adaptation process, and plans for sustaining the intervention once funding ceases. We will also consider the impact of recipient characteristics such as gender (e.g., adolescent girls might not have the same access to food) and socioeconomic status (e.g., impact on health behaviours and outcomes). Qualitative data will be stored in a central depository to facilitate comparative analysis across sites.

### Health economic evaluation

Our health economic evaluation will estimate the resources required to implement the intervention. A bottom-up ingredients-based costing will be conducted to identify the additional resources required to deliver the intervention. Data will be collected retrospectively based on study records and using a costing spreadsheet designed specifically for the study. The local teams will assess the unit costs per activity of the key outputs of the intervention (e.g., number of students counselled). These costs will be presented alongside study outcomes in a cost-consequence analysis and, where appropriate, compared with key outcome measures in a cost-effectiveness analysis (33). We will conduct scenario and sensitivity analyses to illustrate how costs might be affected by implementation strategies, and the potential longer-term impacts beyond study follow-up.

### Analysis plan

We will conduct school-level and individual (cohort) level analysis. We will compare adolescents in the control and intervention schools to determine whether the health literacy intervention is associated with an increase in help-seeking. We will use non-randomised difference-in-difference (DID) analysis (34) to adjust for differences between schools (school-effects) and over time (time effects). The DID design is a quasi-experimental approach which enables the researcher to study causal relationships in settings where randomised control trials are unfeasible or unethical (35). Our outcomes are predominantly proportions (e.g., proportion seeking help), thus we will use a binomial-logistic regression model approach to estimate relative (odds ratios) and absolute (differences in proportions) effects of the intervention. The number of schools is likely too small to reliably estimate school-level random effects, so a mixed model is not considered. We will analyse our universal repeated assessments (administered to all adolescents in intervention and control schools) to determine the effects of the health literacy component on longitudinal mental and physical health measures (baseline, 8, 15, and 22 months). These data will be analysed using an individual level linear model with adolescent random effects, school, time and intervention fixed effects. We will conduct sub-analysis with the cohort of adolescents receiving counselling. The targeted cohort assessments (**Table 2**) will allow us to describe the trajectories of adolescents receiving the counselling component of the intervention to determine whether the counselling has an impact on mental and/or physical health outcomes. For the continuous cohort outcomes (“universal assessment”) the minimum detectable standardised effect size is approximately 0.1 assuming an individual autocorrelation coefficient between time periods of 0.8.

Our sample size calculation is based on the proportion of adolescents seeking help in the control versus intervention groups. While epidemiological findings from LMICs show that approximately 8-10% of youths experience mental health problems requiring intervention, less than 1% receive help (36, 37). Since we are signposting adolescents in the control schools to local services, we anticipate that approximately 5% might seek help. For those receiving the ISOBAR intervention, we predict an increase in the proportion of adolescents who seek care (among those who reach the pre-defined threshold) from 5% (in control arm) to 11-12% (in intervention arm). We are planning to recruit three schools per site. Our sample size calculation assumes an increase in help-seeking of 6-7% in the intervention group compared to the control schools (i.e., 5 to 11-12%). Thus, if we recruit 200 adolescents per school, we will have 80-90% power to detect an improvement of at least 6–7 percentage points. Therefore, we plan to recruit a minimum of 200 adolescents per school per site: 200 x 3 = 600 adolescents per site (Total n = 1,800).

### Effectiveness of the health literacy component

We will estimate the effectiveness of the health literacy component of the intervention in the following ways: (1) through a comparison of the proportion of adolescents seeking help in the intervention versus the control conditions; (2) by assessing impacts on knowledge, attitudes, and behaviour; and (3) by assessing impacts on nutritional (e.g., body mass index, dietary behaviours) and mental (e.g., Strengths and Difficulties Questionnaire) health. These outcomes will be assessed at three follow-up time points: 8, 15 and 22 months.

### Trajectories of adolescents receiving the counselling component

Adolescents forming the cohort referred to counselling will complete assessments at the start of the counselling sessions and at two/three- and 12-month follow-up. Assessments include the Patient Health Questionnaire-9 for depression (38), the Generalised Anxiety Disorder scale-7 (GAD-7) (39), the SDQ follow-up questions to assess the impact of the intervention (40) and the Childrens Global Assessment Scale to assess functioning (41). We will select suitable measures for adolescents receiving counselling for nutritional/weight problems (e.g., food frequency questionnaire, physical activity questionnaire (42)). We will also explore counsellors and adolescents’ experience of the intervention with one-to-one semi-structured interviews.

## Ethics and dissemination

### Ethics

Written informed consent will be collected from adult participants (18 years and over) and from parents or guardians of adolescents under 18 years of age after explaining the study aims, procedure, risks, benefits, and rights. Assent will be obtained from all adolescents under 18 years of age.

The study is conducted in accordance with ethical principles that comply with the Declaration of Helsinki and the International Council on Harmonised Tripartite Guideline for Good Clinical Practice (GCP). Ethical approval for the project has been obtained from the University of Warwick. In addition, each site has obtained ethics approval from their Institutional Ethics Committees (IECs).

### Dissemination strategies

Our study findings will be shared through peer reviewed academic publications, national and international conference presentations, and practitioner and researcher networks. We will maximise the reach of our dissemination with bite sized research reports in layman format, public announcements in communities in LMICs, policy briefings, print and online media, and institutional and professional social media accounts and websites. We anticipate findings from the ISOBAR programme will influence local education and health systems through the implementation of a whole school intervention for mental and nutritional disorders which is accessible to all students regardless of gender, race and socioeconomic status. By implementing and evaluating the proposed intervention in two geographically and culturally distinct LMICs we will establish the contextual effect of the intervention providing evidence and recommendations to inform local, regional, and national policies.

### Patient and public involvement

Stakeholder engagement is a core aspect of the ISOBAR project to ensure effective implementation. Early in the project, teams began developing a network of stakeholders including young people and their caregivers, school leaders and staff, counsellors, healthcare providers, commissioners, policy makers and community groups. During intervention development, the team have worked closely with adolescents, parents and teachers to culturally adapt (and amend following piloting) the intervention to site settings. Teams have worked with relevant educational organisations (e.g., Department of Education, Ministry of Education, Greater Chennai Corporation) and engaged with schools to consider infrastructure, willingness, resources, and timing of exam periods and holidays which could impact the delivery of the intervention.

The parents, teachers, and students provided information about their needs and the topics that they would like addressed in the sessions. The school administrators also discussed the feasibility of delivering the sessions in the school settings. Following the pilot phase, the students and teachers will provide feedback on the content and methodology for delivering the sessions. The teachers and school administrators will comment on the logistics of providing counselling services in the school setting. This process will be used to prioritise the topics to be discussed with adolescents within the broader ambit of adolescent mental and physical health. Stakeholders will be involved at key stages across multiple phases of the project, including intervention development, refinement, and implementation, providing input and feedback to guide adaptation and optimisation of the intervention. This iterative process will support alignment with local needs and enhance the relevance and potential scalability of the intervention.

## Conclusion

This hybrid implementation effectiveness study will contribute to the literature in the following ways: (1) provide detailed data on the cultural adaptation of school-based interventions in LMICs; (2) collate data on pragmatic implementation strategies as relevant to Nigeria and India; (3) provide initial assessment of the effectiveness of a combined school-based physical and mental health intervention for adolescents in LMICs; and (4) explore the potential cost effectiveness of this intervention. It is crucial that we understand the process of cultural adaptation and implementation of school-based interventions in local settings if we are to facilitate sustainability, replication, and scaling-up of these interventions in LMICs (20). Global mental health has only recently started focussing on the costs and benefits of interventions. We will collect real life costs rather than model these hypothetically, which will provide key information for adoption decisions at system and policy level (18).
