# Supplementary material for: A hybrid implementation-effectiveness study of a school-based intervention for promoting health and well-being in low-resource settings: the ISOBAR study protocol
Source: Front Psychiatry. 2026 Jul 14;17:1823889. doi: 10.3389/fpsyt.2026.1823889 (PMC13407776; doi:10.3389/fpsyt.2026.1823889)
Supplement: Supplementary file 1 [file SupplementaryFile1.docx]

Supplementary Material

**ISOBAR programme management**

The ISOBAR team consists of the University of Warwick, UK; McGill University, Canada; University of Ibadan, Nigeria; Centre for Mental Health Law and Policy, Indian Law Society, Pune, India; Schizophrenia Research Foundation, Chennai, India; and the Postgraduate Institute of Medical Education and Research (PGIMER) in Chandigarh, India. The University of Warwick is the sponsor organisation.

A scientific coordinator is responsible for the day-to-day management of the project and supports the principal investigator on the project’s overall delivery. The scientific coordinator, principal investigator and work package leads (based in Nigeria and India) are responsible for the overall management of the study and meet monthly by video conference. The project steering committee (PSC), comprising the programme manager, principal investigator, co-investigators, and work package leads, provides overall strategic direction, ensures good governance, provides scientific input, guides capacity-building activities and provides financial probity when required. An independent external scientific advisory board (ESAB) of international experts monitor and assess the study conduct and progress. Both the PSC and ESAB meet annually with the consortium to assess the scientific quality of the research and provide feedback. The on-the-ground research teams (from Chennai, Gujarat, and Ibadan) meet weekly with the Warwick team by video conference to discuss day-to-day running of the project and progress on project timelines.

**School selection**

At the Gujarat site, the schools will be allocated by the Government Department of Education. All selected schools were public (government) schools.

In Chennai, these schools will be assigned by the Department of Education based on the requirements that were given to them. The requirements included that it be a co-educational school, serve students in grades 6-12, be located in non-contiguous sections of the city ensuring that there was no overlap of students, and have a student population of at least 200 in those target grades.

In Ibadan, the three schools will be purposively selected to reflect the characteristics of low-resource school settings targeted by the intervention.

The selection criteria include:

• Willingness of the school to participate in the study

• Availability of the required school structure (e.g., appropriate student population and administrative support)

• Feasibility of implementing the intervention and conducting follow-up assessments
